# Human papillomavirus self-sampling versus standard clinician-sampling for cervical cancer screening in sub-Saharan Africa: a systematic review and meta-analysis of randomized controlled trials

**DOI:** 10.1186/s13027-021-00380-5

**Published:** 2021-06-19

**Authors:** Hanna Amanuel Tesfahunei, Michael Solomon Ghebreyesus, Dawit Getachew Assefa, Eden Dagnachew Zeleke, Joan Acam, Michele Joseph, Emnet Getachew, Violet Dismas Kajogoo, Delayehu Bekele, Tsegahun Manyazewal

**Affiliations:** 1 Addis Ababa University, College of Health Sciences, Center for Innovative Drug Development and Therapeutic Trials for Africa (CDT-Africa), P.O. Box 9086, Addis Ababa, Ethiopia; 2 Hager Biomedical Research Institute, Asmara, Eritrea; 3 Department of Public Health, Africa Medical College, Addis Ababa, Ethiopia; 4 Department of Nursing, College of Health Science and Medicine, Dilla University, Dilla, Ethiopia; 5 Department of Midwifery, College of Health Science, Bule-Hora University, Bule-Hora, Ethiopia; 6 Pope John’s Hospital Aber, Oyam District, Uganda; 7 Arsi University, Asella, Ethiopia; 8 Mafia District Hospital, Mafia Island, Tanzania; 9 Department of Obstetrics and Gynecology, Saint Paul’s Hospital Millennium Medical College, Addis Ababa, Ethiopia

**Keywords:** Human papillomavirus (HPV), Self-sampling, Cervical cancer screening, Sub-Saharan Africa, Randomized controlled trial, Systematic review and meta-analysis

## Abstract

**Background:**

Human papillomavirus (HPV) infection remains a major health threat in sub-Saharan Africa (SSA). HPV self-sampling could help find and treat cervical cancer at an early stage. We aimed to evaluate the effectiveness of HPV self-sampling over the standard health facility-based clinician-sampling for cervical cancer screening through a systematic review and meta-analysis of available randomized controlled trials.

**Method:**

We searched PubMed, Cochrane Central Register of Controlled Trials, ClinicalTrial.gov, and the WHO Global Health Library for articles in SSA published as of 31 May 2020. We followed the Preferred Reporting Items for Systematic Review and Meta-Analysis Protocols (PRISMA-P) 2015 guidelines for the design and reporting of the results. We included randomized control trials that compared HPV self-sampling with the standard of care. The primary endpoint was uptake of cervical cancer screening service. The secondary endpoints were linkage to care, acceptability, screening frequency, and adverse events. We used RevMan V.5.3 software for statistical analysis. We computed random-effect model to provide pooled estimates of available data and I-squared (I^2^) test to assess heterogeneity.

**Result:**

Of 77 citations, we included four trials from Nigeria, Ethiopia, Kenya, and Uganda, encompassing 8200 participants with age ranging from 25 to 65 years. The pooled analysis showed significantly higher uptake of cervical cancer screening in women who used HPV self-sampling (risk ratio [RR] 1.72, 95% CI 1.58–1.87; *p* = 0.01), while this had a considerable heterogeneity as explained by subgroup analysis. Uptake was higher in women who were offered sampling kit at home or work (RR 2.05, 95% CI 1.80–2.33) and those who’s kit was mailed to or invited to a nearby health center (RR 1.65, 95% CI 1.58–1.72, I^2^ = 0%) than those screened with the standard of care. There was no difference between the two groups in the rate of linkage to care of positive cases (RR 1.30, 95% CI 0.90–2.74, I^2^ = 91%). HPV self-sampling was acceptable and easy to use. None of the trials compared the frequency of screening or adverse events.

**Conclusion:**

HPV self-sampling is an effective and feasible alternative to the standard health facility-based clinician-sampling for cervical cancer screening in SSA. It could improve the uptake of cervical cancer screening and harness the global strategy towards elimination of cervical cancer by 2030.

## Introduction

Cervical cancer is the third most common cancer in women globally and the second most common malignancy in developing countries. Over 500,000 women were diagnosed with cervical cancer in 2018 and 311,000 of those died from the disease [1]. More than 85% of the deaths due to cervical cancer occur in low-middle income countries (LMICs). Cervical cancer presents a significant public health threat to women on the African continent; out of the top 20 countries with the highest-burden worldwide, 19 were African countries [2]. Potentiated by HIV infection this disease is steadily increasing in sub-Saharan Africa (SSA), with more than 75,000 new cases and 50,000 deaths yearly [3]. It is estimated that cervical cancer will kill more than 443,000 women yearly worldwide by the year 2030, most of them in SSA. This surge in the incidence of cervical cancer cases in Africa could deter the progress made by African women in reducing maternal mortality and longevity [4]. Nevertheless, cervical cancer is a potentially preventable and curable disease if diagnosed and treated early. Poor access to prevention, screening, and treatment contributes to 90% of deaths hence intervention strategies to eliminate cervical cancer as a public health concern should be urgently implemented [3]. The majority of cervical cancer cases (99%) are linked to infection with high-risk human papillomaviruses (HPV) [2]. Currently, HPV is the most common sexually transmitted infection and it is estimated that 80% of women are infected with this virus at some point in their lifetime [5].

Cervical cancer brings about a significant threat to Sub-Saharan women and the conventional way of screening (VIA, clinician collected HPV and Pap smear) women at risk has not been practical nor has it been accessible for the majority of women leaving in SSA [6]. In low-resource settings, various barriers to implementing cervical cancer screening programs exist. These include lack of trained personnel, lack of laboratory supplies, lack of laboratory infrastructure, socio-religious and cultural barriers to pelvic examination, and limited physical access to patient populations [7]. An alternative screening method is in need to address access challenges as well as personal barriers in sub-Saharan women.

According to the World Health Organization (WHO), HPV DNA screening is the recommended screening method in LMIC [6], and HPV self-sampling is found to be more acceptable as it overcomes the personal barriers such as shame, embarrassment, and reluctance in letting a clinician see or touch their genitals [8, 9]. Self-sampling for HPV has already been shown to be a convenient and cost-effective method of cervical cancer screening among the hard-to-reach population [10]. Studies conducted in Africa reported a higher HPV diagnostic concordance between self-collected and physician-collected HPV specimens [9–11]. Studies reported a higher linkage to care and follow-up of women who tested HPV-positive in a self-obtained sample [12, 13]. The socio-cultural barriers to HPV screening that were documented in LMICs studies apply in SSA [11].

Based on a systematic review and meta-analysis of 29 RCT and four observational studies conducted by the WHO, women who used HPV self-sampling were twice likely to get screened and benefit from the service without any negative effect on linkage to care [6]. However, majority (93%) of the study participants were from high-income countries and hence are not representative of LMIC. There is a need to conduct a systematic review and meta-analysis focusing on LMICs, particularly the SSA.

Therefore, We aimed to evaluate the effectiveness of HPV self-sampling over the standard health facility-based clinician-sampling for cervical cancer screening through a systematic review and meta-analysis of available randomized controlled trials.

## Methods

We followed the Preferred Reporting Items for Systematic Review and Meta-Analysis Protocols (PRISMA-P) 2015 guidelines (https://www.google.com/search?rlz=1C1CHBF_enUS714US714%2Cprisma+guidelines+for+systematic+reviews+pdf, [14]) for the design and reporting of the results.

### Eligibility criteria

We PICOS (participants, interventions, comparison, outcomes, and study designs) description model to formulate participants’ eligibility criteria.

- **Participants**
  - Women
  - Resident of one of the countries in SSA,
- **Intervention**
  - HPV self-sampling for cervical cancer screening
- **Comparator**
  - Standard health facility-based clinician-sampling for cervical cancer screening
- **Outcome**
  - Primary outcome
    - Uptake of cervical cancer screening
  - Secondary outcomes
    - Acceptability
    - Frequency of screening
    - Adverse event
    - Linkage to care
- **Study design**
  - Randomized controlled trial

### Data sources and search strategy

We searched PubMed, Cochrane Central Register of controlled trials, ClinicalTrial.gov, and WHO Global Health Library for articles in SSA published as of 31 May 2020. We applied MeSH (Medical Subject Headings) and text words (synonyms and related terms) were to search the terms. This included a combination of MeSH terms for SSA, HPV, and self-sampling or self-collection, with Boolean operations used in between search terms. We came up with a search term: (“Human Papilloma Virus” AND “Self-collected OR Self-sampling OR Self-obtained” AND “Sub-Saharan Africa”), filters were put on Randomized controlled trials, and Clinical trials. The term “Retracted publication” was included in the search terms to exclude retracted articles. Reference lists of the relevant studies found were also assessed to further search for relevant studies.

### Study selection

**Fig. 1:**
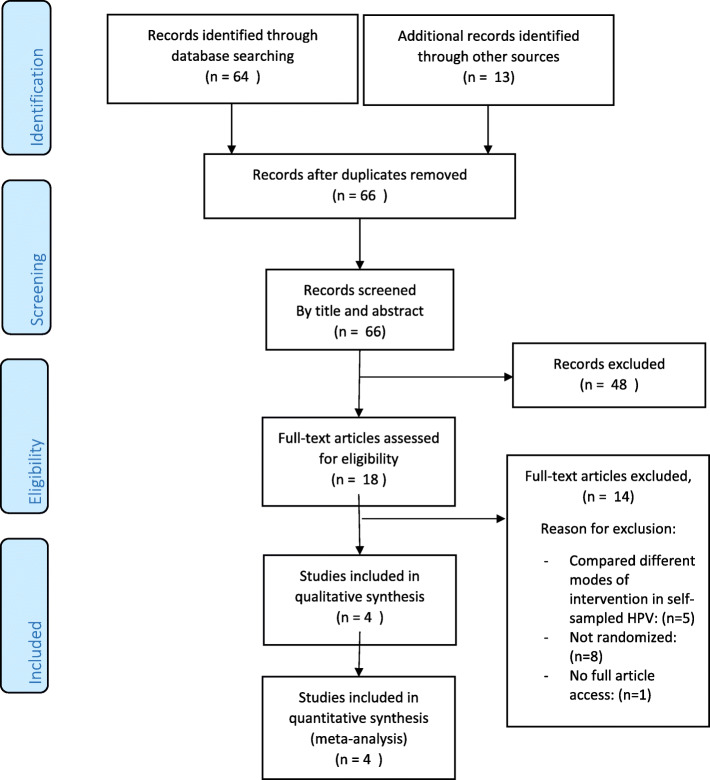
PRISMA diagram of the study

After initial title–abstract screening, full-text articles were obtained of all potential studies and relevant articles were retrieved and assessed further. Two reviewers independently assessed all full-text articles for study inclusion eligibility set above and resolved differences with a third responsible author through consensus. Figure 1 summarises the design that we used to report the study result in line with the PRISMA-P 2015 guidelines.

### Data extraction

Two reviewers independently extracted data and conducted the quality assessments and resolved differences with a third responsible author through consensus. Standardized data extraction forms included fields for study location, population setting (rural, urban), population characteristics, description of the intervention and control used, type of sampling device used, study design, sample size, reported outcomes, results, and inclusion and exclusion criteria used in each study. The number of participants randomized and the number analyzed in each treatment group for each outcome was also collected.

### Assessment of risk of bias

The risk of bias was assessed using the Cochrane Collaboration’s tool (ROB version 2) [15]. Two independent authors reviewed it and resolved disagreement with a third responsible author. Domains such as sequence generation, allocation concealment, blinding of participants/outcome assessors, missing outcome data, selective outcome reporting, and other potential bias were assessed for risk of bias. The ratings were “high risk”, “unclear risk”, and “low risk”. A study with at least one domain with a high risk of bias was considered a “high-risk study” and a study with all its domains with low-risk bias was considered a “low-risk study”. The risk of bias assessment was performed on the Rev-Man version 5.4 software.

### Statistical analysis

The primary outcome of interest was uptake of cervical cancer screening service that is defined as the proportion of those offered HPV testing or other screening methods who accepted and completed screening. And we combined the authors’ reported data on screening participation, attendance, response, and compliance on both the interventional (HPV self-sampling) group and the control group (using a standard of care screening). For the outcome linkage to clinical assessment or treatment following a positive self-test result and a positive diagnosis for HPV by a healthcare provider, the proportion who reach this next stage of management was taken. Where multiple studies reported the same outcome of interest, we conducted a meta-analysis using random-effects models to generate pooled relative risk (RR) with a 95% confidence interval using the Rev-Man 5.4. Intention-to-treat data was used as all the studies analyzed and reported intention-to-treat. Publication bias could not be assessed as the number of studies was too low. However, the risk of bias across studies was assessed with selective reporting within studies. Heterogeneity was assessed using I-squared (I^2^) statistics and results were interpreted using the Cochrane Handbook for Systematic Reviews of Interventions, Version 6.1 [16]. Subgroup analysis was done to investigate the source of heterogeneity in the subgroup; “timing of outcome data collection”. We conducted a sensitivity analysis by excluding and including the study to check if there is a significant impact using Rev-Man 5.4 on risk of bias and to check the missing effects.

### Ethical considerations

This study did not require ethical approval and informed consent. Because participant’s data for this systematic review and meta-analysis were exclusively extracted from published studies.

## Results

### Study selection

The electronic database searching retrieved 64 citations. Secondary searching such as reference lists brought additional 13 citations (Fig. 1). After removing duplicates, there were 66 unique citations. After the initial screening of titles and abstracts, 18 citations remained for full-text review. Of these, four eligible studies were included in the review [17–20].

### Study characteristics

Table 1 presents the summary characteristics of the four included articles. The studies were from Ethiopia [17], Nigeria [18], Kenya [19], and Uganda [20]. The studies included a total of 8200 participants, with individual study sample sizes ranging from 400 to 4944 and the age group ranged from 25 to 65 years. The majority of the women in the studies fell under the age group 30–39 years.

**Table 1:** Characteristics of included RCTs

Author, Year	Characteristics	Sample size (in each group)	Intervention information	Design	Inclusion criteria	Exclusion criteria	Outcomes
Gizaw et al. 2019 [17]	Ethiopia, Rural and Urban Population, Women around Butajira vicinity Age: 30–49	2356: intervention arm: 1213 control arm 1143	**Intervention**: HPV self-sampling in the primary health care unit at their vicinity in a private area under active **supervision** by a trained health professional **Prior Sensitization** on cervical cancer and HPV and instruction on self-sampling given. **Device**: Evalyn Brush (Rovers) **Specimen:** not specified **Control**: Butajira hospital for VIA screening	Cluster RCT	Age: 30–49 Never been screened before	Women were excluded if they were pregnant, actively bleeding, had a previous hysterectomy, and refused to give consent before the screening.	Uptake and Linkage to care
Modibbo et al. 2017 [18]	Nigeria. Semi-urban. Women residing in Karu Age: 30–65.	400 intervention: 200 control: 200	**Intervention**: HPV self-sampling kit directly mailed to home address with prepaid return envelope (or could drop off completed kit at designated collection points in community or at the hospital). **Unsupervised**. Collected at home. **Prion sensitization;** health education on cervical cancer, its risk factors**.** **Device**: Dry flocked Swab. **Specimen**; Cervicovaginal **Control**: clinician- Collected HPV testing appointment at hospital clinic.	RCT	Inclusion criteria were women aged between 30 and 65 years, living or working in Karu who do not plan to move out of the community over the next 6 months.	Pregnant, planning to relocate within six months, HIV positive, had unexplained cervical bleeding, history of hysterectomy, mental illness or cervical cancer from the study.	Uptake of HPV testing services and Acceptance.
Moses et al. 2015 [20]	Uganda. Semi-urban. Women residing in Kisenyi near Kampala Age: 30–65.	500 intervention: 250; control 250.	**Intervention**: HPV self-sampling. **Instruction**, how to self-collect a vaginal specimen using a standard script and diagram by the outreach workers. **No prior sensitization Unsupervised**. At work place or home up on recruitment. **Device**: Dacron swab. **Specimen;** Cervicovaginal **Control**: VIA Screened in Kisenyi healthcare center	RCT	Included if between 30 and 65 years of age, lived or worked in Kisenyi, and had access to a mobile telephone. Who had an intact uterus and cervix.	Excluded if they had a previous hysterectomy or cervical cancer, if they did not meet the eligibility criteria or if they were unable to give consent	Uptake of screening and Linkage to care
Megan et al. 2018 [19]	Kenya, Rural setting, women residing in Migori, western Kenya Age, 25–65	4944 intervention arm = 2898 control = 2046	**Intervention**: HPV self-sampling screening was offered in tents around villages Under supervision of community health volunteers (CHV). Self-screening **instruction** given by CHV **Prion sensitization** on cervical cancer and HPV. **Device**: not mentioned **Specimen**: Vaginal **Control:** Clinician- collected HPV screening was offered at government health facilities in Migori.	Cluster RCT.	Communities were considered eligible if they had at least one government health facility with the capacity to offer HPV testing, received support from community leaders for community outreach and/or health campaigns, offered access to health centers via a maintained transportation route and were not bordering other study sites to limit contamination between arms (buffer zones).	Urban settings were excluded from the study.	Uptake of screening, acceptance and linkage to care.

The articles were published between 2015 and 2019, with the latest published in Ethiopia. The studies had different settings: Gizaw et al. [17] enrolled from urban and rural, with the majority (86%) coming from a rural setting; Modibbo et al. [18] and Moses et al. [20] enrolled from semi-urban settings that were both near the city capital of their countries but with impoverished lifestyle; and Megan et al. [19] enrolled only from rural settings.

Megan et al’s study was conducted in a setting with the highest prevalence of HIV (15%) in Kenya and a higher prevalence of HPV among HIV-positive women. Women living with HIV were more likely to prefer the HPV standard of care sampling than HPV self-sampling (38% vs 25%). This was similar in Moses et al. [20], where women who were HIV positive and with chronic disease were more likely to attend standard of care. Gizaw et al. and Megan et al. first divided their study villages into community/ kebele clusters and randomized these clusters to either interventional group or control group. Community mobilization was conducted in each cluster by health extension workers (Gizaw et al) or community health volunteers (Megan et al). The clusters in the interventional arm were given self-sampling instructions while those in the control group were informed to go to the near-by health facility for clinician-sampling. In Modibbo et al., 2017 [18], they invited all women 30–65 to the king’s palace and gave information on the research, on cervical cancer and risk factors. And after consenting, the women were assigned to the intervention and control groups. In women randomized to the intervention arm, HPV self-sampling kit along with instructions was directly mailed to their home address, while those assigned to the hospital group were given appointments for the clinic. Moses et al. [20] was a pilot RCT; study outreach workers approached women in their homes or places of work, invited to participate, in the study, and randomized at the spot. Those women randomized to the self-sampling group were given Dacron swab and instructed on how to use it and they provided specimens in a private room immediately at the place where they were recruited. And those who were randomized to the VIA arm were scheduled a date to attend the health unit to undergo VIA for screening. Participants were provided a reminder phone call the day before their scheduled visit.

All the studies compared an interventional group with HPV self-sampling method with a control group with the standard of care (with VIA in Gizaw et al. and Moses et al. and with clinician collected HPV in Megan et al. and Modibbo et al. Swab and brush collection method was reported among the self-sampling arm of the studies, Modibbo et al. used dry flocked swap, Moses et al. used Dacron swab while Gizaw et al. used Evalyn brush collection method for HPV self-sampling. The collection method was not reported in Megan et al. Cervicovaginal specimen was collected in Modibbo et al. and Moses et al., while vaginal specimen was collected in Megan et al.

### Random sequence generation (selection bias)

**Fig. 2:**
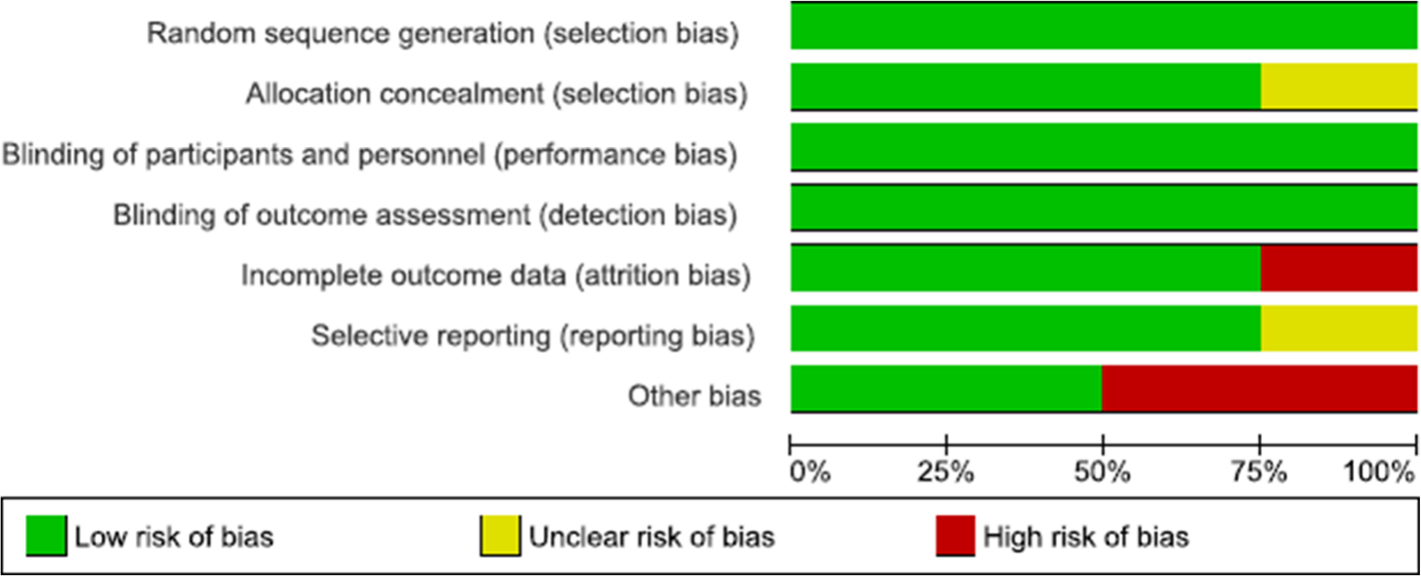
Risk of bias graph

**Fig. 3:**
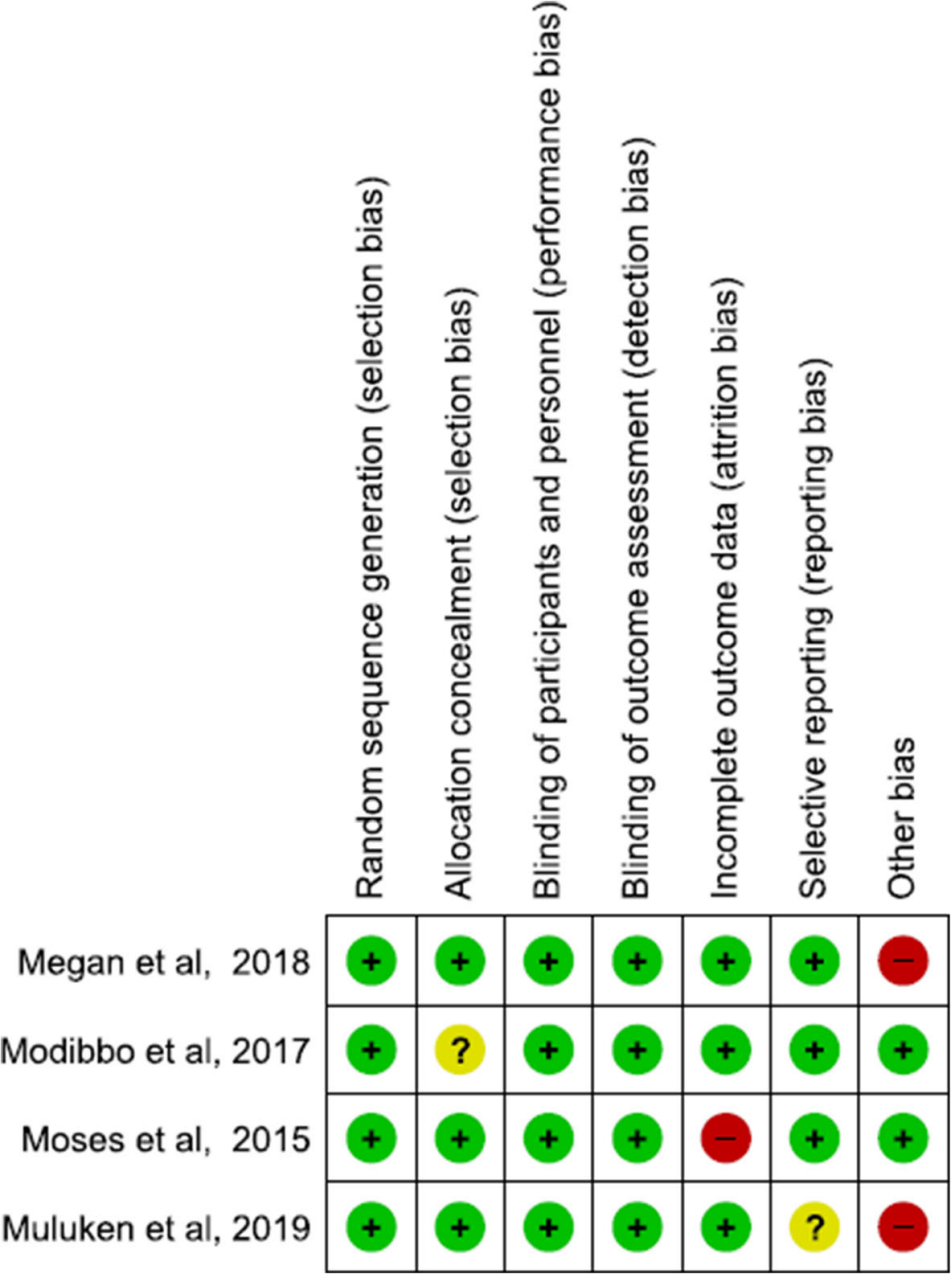
Risk of bias summary

All the included studies had a low-risk of bias in this domain. Included studies generated their random sequence using different software such as Stata/MP (Megan et al), random number tables from SAS 9.3 (Megan et al) and Moses et al) and research random software (Gizaw et al) (Figs. 2 and 3).

### Allocation concealment (selection bias)

Three studies (Gizaw et al., Moses et al., and Megan et al) were judged to have a low-risk of bias on allocation concealment. Moses et al. mentioned that allocation was recorded on cards, which were concealed in an envelope and kept in a locked cabinet. And Gizaw et al. and Megan et al. both conducted cluster RCT, and they randomized their clusters at the same time; therefore, allocation concealment was not an issue and hence they were labeled as low-risk of bias. Megan et al. did not mention their attempt to conceal the allocation and hence was labeled as unclear-risk.

### Blinding of participants and personnel (performance bias)

Due to the intervention of interest, blinding the participants was not possible; however, the outcomes measured were not likely to be biased by the absence of blinding as uptake and linkage to care were measured as the number of kits sent to laboratory and medical records and not by self-reporting.

### Blinding of outcome assessment (detection bias)

Blinding of outcomes assessors was also not possible given the type of intervention and this also is not likely to cause bias as outcomes were measured as the number of kits sent to lab and medical records.

### Incomplete outcome data (attrition bias)

Three of the studies (Megan et al., Gizaw et al., and Modibbo et al) had a low-risk of bias in this domain as they had no missing data. But Moses et al. had 51% missing individuals in the second outcome assessment of linkage to care and was judged as high-risk of bias.

### Selective reporting (reporting bias)

All four studies reported outcomes that were pre-specified in their protocol and had low-risk of bias.

### Other bias

Both Gizaw et al. and Megan et al. that conducted cluster RCT enrolled participants after randomization of clusters to intervention and control arms. Therefore this can lead to recruitment bias as they were recruited after knowledge of which cluster was intervention group and which was control. And hence they were labeled as “high risk of bias”.

### Results of individual studies

Results on the outcomes; screening uptake and linkage to care are summarized in Table 2.

**Table 2:** Summary result of individual studies

Uptake	Self Sampling HPV						Standard of care					
	n in arm	uptake	% uptake	*n* = +veHPV	linkage to care	%	n	uptake	% uptake	*n* = +veHPV	linkage to care	%
Giza et al. 2019 [17]	1213	1020	84.10%	144	122	85%	1143	575	50.50%	22	5	23%
Modibbo et al. 2017 [18]	200	185	93%	NA	NA		200	113	56%	NA	NA	
Moses et al. 2015 [20]	250	248	99.20%	73	33	45%	250	121	48.40%	16	12	75%
Megan et al. 2018 [19]	2898	1739	60%	567	222	39%	2046	757	37%	476	150	31%

### Acceptance of screening

Two studies reported outcomes on acceptance of screening method Modibbo et al. and Megan et al. In Modibbo et al., most of the women (95.2%, 177/185) found the self-sampling device easy to use and 83.2% (154/185) reported that they would prefer self-sampling as a future screening option than health facility-based sampling. In Megan et al., 99.1% (2872) said they would test again via self-collection and 99.4% (2881) said they would recommend testing via self-sampling to a friend.

### Synthesis of results

#### Uptake of self-sampled HPV screening

**Fig. 4:**
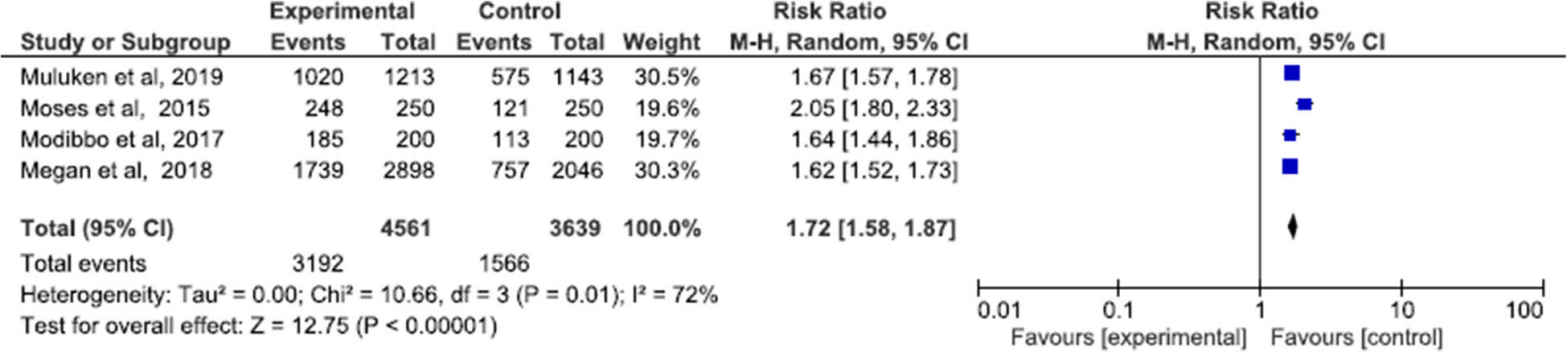
Meta-analysis uptake of self-sampled HPV screening

All the studies included in the review reported uptake as the proportion of those offered HPV testing or other screening methods who accepted and completed screening. Self-sampling HPV in intervention group and standard of care (either clinician collected HPV or VIA) in control group were compared in assessing uptake. A meta-analysis of the four RCTs shows that the uptake of women using self-sampling HPV as method of cervical cancer screening is 72% higher than those using the standard of care (RR: 1.72, 95% CI 1.58 to 1.87, I-squared: 72%) (Fig. 4). However we cannot rely on this pooled result as the synthesis showed substantial heterogeneity 72% which was then explained by subgroup analysis explained below.

#### Uptake: sub-grouped by timing of outcome data collection

**Fig. 5:**
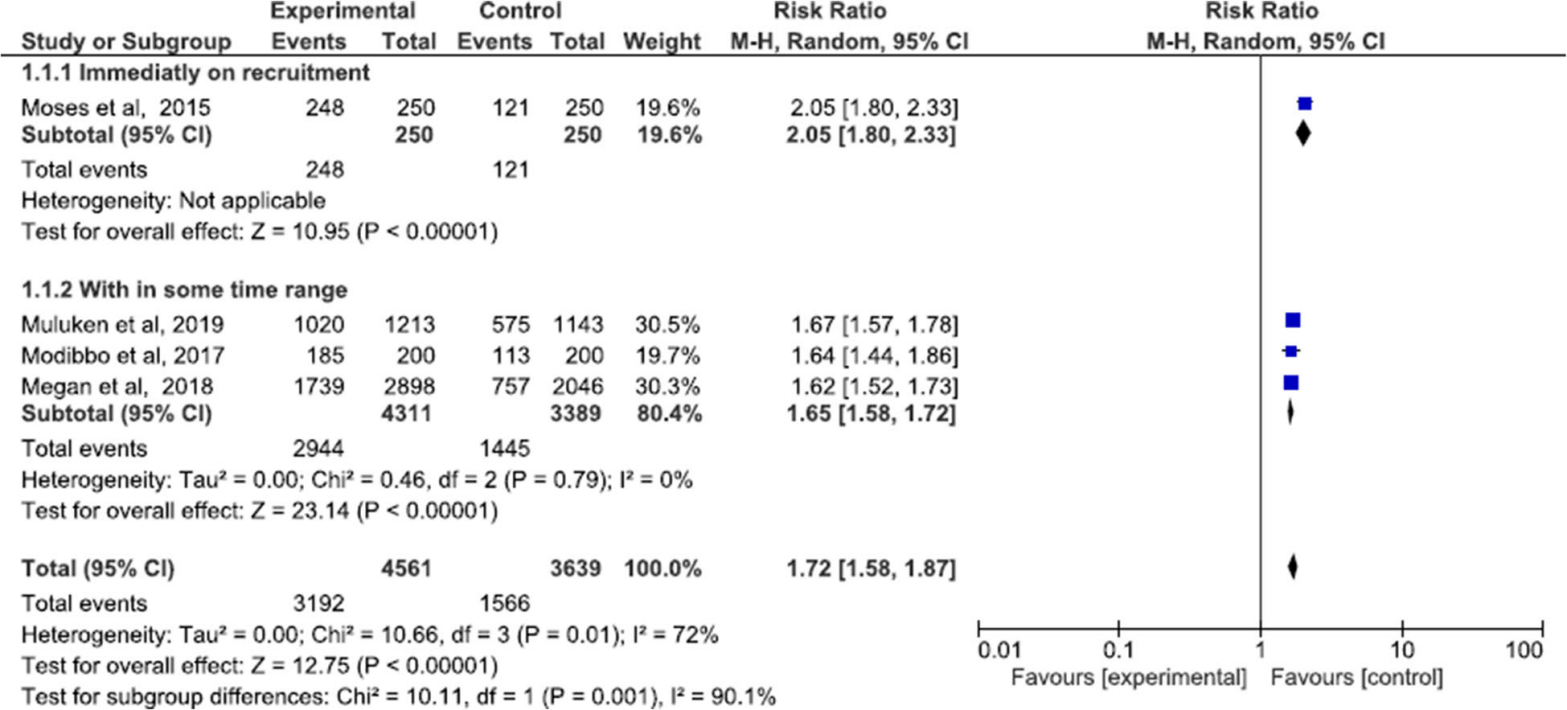
Meta-analysis uptake: Sub-grouped by timing of outcome data

Outcome collection time ranged from immediately upon recruitment to 2 months. In Megan et al. and Gizaw et al., after subjects consented and were randomized, they were told to either go to the nearby health care (for self-sampling HPV group) or the hospital (for the standard of care). Whereas for Modibbo et al., subjects were send sampling kits via mail (for those randomized in self-sampling HPV group) or appointed to go to the hospital (for the standard of care group). However, this was different in Moses et al., where women randomized to the self-sampling group were given sampling kits upon recruitment and those randomized to standards of care were appointed to the hospital. To investigate whether this is the explanation to the heterogeneity that has occurred we sub-grouped the studies based on follow-up time as Moses et al., “immediately on recruitment” and as for the Modibbo et al., Megan et al., and Gizaw et al. as “within some time range”. And the meta-analysis of the subgroup shows a significant difference between subgroups (*p* = 0.001) and in those women in HPV self-sampling group whose samples were taken immediately upon recruitment, uptake was 2.05 (RR = 2.05, 95% CI 1.80 to 2.33) times higher in the HPV-self sampling group than the standards of care. And on those studies with HPV self-sampling groups who gave samples within some time range after recruitment, uptake was 65% (RR = 1.65, 95% CI 1.58 to 1.72, I^2^ = 0%) higher on the HPV self-sampling group. And the heterogeneity was 0 %, therefore this was the factor or explanation to the heterogeneity that occurred (Fig. 5).

#### Uptake: subgroup by supervision

Subgroup analysis was performed to see if there is a difference on the presence or absence of a supervising body; however, results showed that there no significant difference between the studies with and without supervision.

#### Linkage to care

**Fig. 6:**
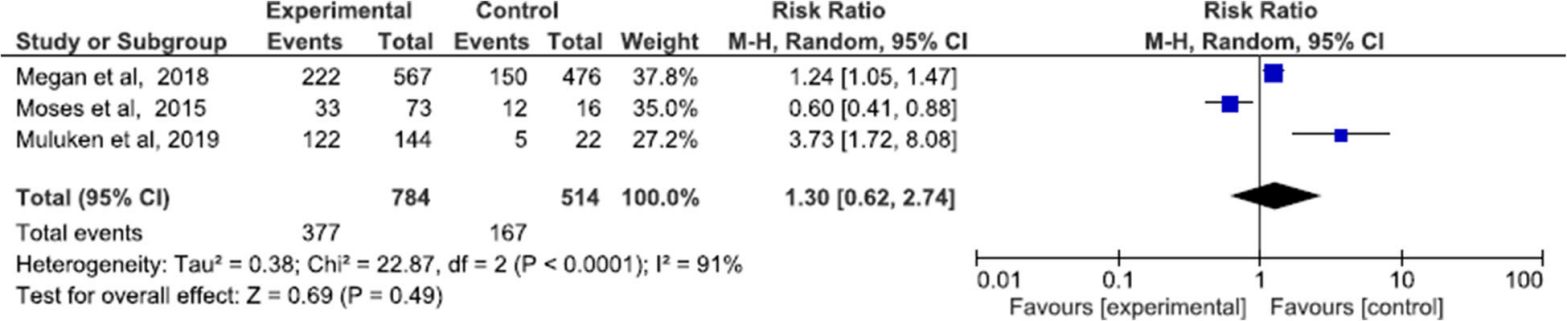
Linkage to care

Three studies reported the proportion of those women who got a positive result and reached a health center for further treatment or recommendation. Megan et al., Gizaw et al., and Moses et al. The meta-analysis found no difference in the rate of linkage to care among women who received a positive screening result between arms (RR = 1.30, 95% CI 0.90 to 2.74, I^2^ = 91%) (Fig. 6).

#### Acceptability of the screening methods

None of the included articles compared the acceptance of screening methods between the two arms and hence no meta-analysis was done. However, two studies have reported acceptance on HPV self-sampling arm and it is summarized in narration in the results of the individual studies.

#### Frequency of cervical cancer screening

No study reported comparative data on the frequency of cervical cancer screening.

#### Adverse events

No study compared outcomes relating to adverse events.

### Risk of bias across studies

We could not assess publication bias as our studies were below 10 (4 studies).

### Additional analysis

Subgroup analysis was performed and the results are in the above section under uptake. Sensitivity analysis was performed to check the robustness of results by removing and adding the high risk of bias studies and there was no significant change. Moses et al. had significant missing values on measuring linkage to care and this study was removed to check if it affects the pooled outcome, however the meta-analysis was still not significant.

## Discussion

Cervical cancer brings about a significant threat to Sub-Saharan women and the conventional way of screening (VIA, clinician collected HPV and Pap smear) women at risk has not been practical nor has it been accessible for the majority of women leaving in Sub-Saharan Africa [6, 21]. An alternative screening method is in need to address access challenges as well as personal barriers in Sub-Saharan women. HPV self-sampling is found to be more acceptable as it overcomes the personal barriers such as shame, embarrassment, and reluctance in letting a doctor see or touch their genitals [8].

Although there was inter-study heterogeneity, screening uptake was higher in the group that used HPV self-sampling. And after subgrouping and exploring heterogeneity, it was found that women were more likely to get screened when the sampling kit is offered at their home or workplace by health workers than those who were mailed or given the responsibility to come to a nearby clinic and get screened. The meta-analysis showed no significant difference between HPV self-sampling groups that were supervised and those unsupervised, the uptake was the same for both groups. There was no significant difference on linkage to care between HPV self-sampling group and standard of care group. Linkage to care was high in Gizaw et al.; however, this was not the case for Moses et al. and Megan et al. Although it is encouraging that linkage to care is not lower than the standard of care, the overall low rate of medical follow-up after positive screening result is of concern. All the included studies did not report the frequency of screening or harm/adverse event related to screening. However, given that cervical screening is recommended at most every 5 years, it might be difficult for RCT studies to have long term studies assessing frequencies of screening. This review adds on evidence from a systematic review in Africa showing concordance of HPV-self sampling and physician collected samples [9]. The results of this review are in agreement with previous systematic review and meta-analysis, showing higher uptake and no difference of linkage to care [6, 21, 22].

Acceptability of HPV self-sampling was reported by Modibbo et al. and Megan et al. And most of the women found the self-sampling device easy to use, said they would prefer self-sampling in the future than hospital screening and that they would recommend testing via self-sampling to a friend. Another review also shows that HPV self-sampling is highly accepted as the participants found it easy and convenient [22]. This was also reflected in observational studies [23–30]. Different observational studies conducted in sub-Saharan Africa assessed acceptance of self-sampling using different questions; ease of use of the device, the comfort, privacy, whether they would recommend to a friend, if they felt relaxed while using it, and if they felt confidence on doing the test properly. And a significantly higher number of women gave positive feedback on favor of self-sampling, however, when it comes to their confidence of doing the test properly higher number of women reported that they would feel more comfortable of the results if it was performed by a clinician [23–26, 30]. This was similar to the study done in Botswana, although the number was significantly high in favor of self-sampling when it comes to ease, comfort and preference, this was not the case when the women were asked if they felt confidence on doing the test properly [28]. Although a statistically significant majority of the participants preferred self-sampling, a proportion of them were concerned regarding their ability to collect the sample correctly and trusting the physician sampling. Their reasoning lies with the trust of the clinician’s skills and their low confidence on the performance. This was also supported by a studies conducted in rural Ethiopia, Cameroon and South Africa, where the participants provided similar reasoning to their concern, and that is their trust in the doctor’s expertise and finding it reliable [23, 24, 29]. A number of observational studies tried to examine the association of this preferences to education and age, although there was no significant association to age, they found a significant association with education stating that the women who reported less confidence towards their sample are the ones with lower educational level [23, 28, 29]. This may indicate that provision of instructions appropriate to each study population, literate and illiterate, on HPV self-sampling is essential to increase women’s confidence in their ability to perform the test correctly and achieve a high uptake of HPV screening in sub-Saharan countries.

This study has some limitations. Including only randomized controlled trials was both our strength and limitation. It was our strength as it had a higher degree of evidence however it was limiting as there are a lot of observational studies that can address our research question. Two studies Megan et al. and Gizaw et al. that conducted cluster randomized trial recruited after randomizing and this might have led to recruitment bias and favored the intervention, hence more studies with more degree of evidence need to be conducted. The strength of this study is that it included different types of population (HIV women, women with no prior screening) in different settings (different countries, urban, rural semi-urban). Our confidence in the findings of this study is supported by our large number of participants, however, there were only four out of the 46 sub-Saharan countries included in this study. Therefore, more studies need to be conducted in different sub-Saharan countries to assess uptake and different kit dissemination approaches. This study has shown an increase in uptake of screening with HPV self-sampling, but researches need to address which support material (health education, video illustrations or in-person training, or supervision), which of these components can increase uptake among different populations such as the vulnerable (e.g. women with HIV or those with multiple sex partners), older women, illiterate women and others. More researches are also needed to assess the mechanism to increase linkage to care of the self-sampling women.

## Conclusion

HPV self-sampling is an effective and feasible alternative to the standard health facility-based clinician-sampling for cervical cancer screening in SSA. It could improve the uptake of cervical cancer screening and harness the global strategy towards elimination of cervical cancer by 2030. However, further studies are needed that compare cost-effectiveness of the two sampling methods in SSA.

## Data Availability

The dataset supporting the conclusions of this article is included within the article.

## Abbreviations

LMIC: Low and middle income countries
SSA: Sub-Saharan African
VIA: Visualization with acetic acid
HPV: Human papillomavirus
hr-HPV: High risk human papilloma virus
WHO: World health organization
DNA: Deoxyribonucleic acid
RNA: Ribonucleic acid
HIV: Human immunodeficiency virus
RCT: Randomized controlled trial
PICOS: Participant, intervention, comparative, outcome, study design
